# The Book World of Medicine and Science

**Published:** 1902-11-08

**Authors:** 


### The Book World of Medicine and Science.

A Manual of Surgery for Students and Practitioners. By William Rose, M.B., B.S.Lond., F.R.C.S., and Albert Carless, M.S.Lond., F.R.C.S. Fifth edition. (London: Baillière, Tindall and Cox. 1902. Price 21s. net.)

Yet another edition of Rose and Carless within a year of the publication of the last. This is indeed phenomenal success. Yet it is well deserved, for probably no manual of surgery has so exactly hit the requirements of the student and the practitioner as this has done. We have so recently reviewed this book that we might content ourselves with saying that in the present edition it has been thoroughly brought up to date, and that in various ways the subject-matter has been so re-arranged as to improve the balance of its several parts. The book has, however, taken such a position that it will be interesting to refer briefly to its teaching in regard to a few points which have of late been more or less discussed. Turning to the first chapter the statements made in regard to asepsis and the use of antiseptics are of some importance in view of the wide, indeed general, acceptance of asepsis as the ideal. After a careful account of the facts of bacteriology, a full description is given of the antiseptic method of operating as practised at King's College Hospital, in the course of which we note that "during the operation the wound may be occasionally irrigated with a 1 in 40 carbolic solution, or with corrosive sublimate (1 in 2,000); but such is not always advisable, as it increases the amount of subsequent oozing, and is really unnecessary if one is certain as to the aseptic condition of everything employed." Further on it is laid down that "the rule of practice which we are now endeavouring to establish is *the strictest antisepsis for external parts, but asepsis for the the interior of the wound*; one cannot always be certain, however, that this ideal has been attained, and then antiseptic irrigation may be resorted to." Speaking of "aseptic surgery," it is stated that this method requires more attention to details than does the antiseptic method, and that where assistants are constantly changing, as in a large teaching hospital, and where many hands are engaged in the work, there is much greater risk of failure. "It is only natural," it is added, "that we who have had the privilege of working with Lord Lister, and have seen the excellent results following the intelligent use of antiseptics, should still to a large extent cling to that line of practice, which certainly can be carried out with more precision under all circumstances, both in private and hospital, than the other plan, the objects of which may at any moment be defeated by some slight inadvertence or oversight." This, we think, fairly represents the present position of the question; asepsis when it can be assured, but antiseptics, even to the wound, whenever there is any doubt as to the aseptic conditions. In the treatment of adenoids we notice two things: first, that "as a general rule the child should be anæsthetised with chloroform," and, second, that the use of Löwenberg's forceps is recommended, in both of which courses the authors differ from many authorities. As to the treatment of appendicitis, the authors say that in the milder forms, "where the temperature does not run above 101°, and the symptoms are not severe, all that is required in the majority of instances is to put the patient to bed and apply fomentations locally; the lower bowel should be emptied by an enema, and if it seems likely that there is an accumulation of irritating fæces within the intestines, a dose of castor oil or of calomel may be administered." But medical treatment can never be depended on and, unfortunately, only too many lives have been sacrificed through an unwillingness to call in a surgeon except at the last moment. "Personally, we are distinctly in favour of early operation, and the general rule (to which, of course, there are exceptions) which we should suggest as justifiable, is that *if in spite of suitable rest and medical treatment the symptoms, both general and local, are not commencing to abate at the end of* 48 *hours, operation should be undertaken*." Throughout the book there is constant evidence of careful thought, both in regard to matter, arrangement, and condensation. The latter is an important consideration for the student, but it is to be noted that condensation has not been allowed to interfere with the definiteness and the clearness of the teaching. It is a work to which the practitioner may with advantage constantly refer, but it is pre-eminently and before all a teaching-book, and as such it probably has no equal at the present day.

A Manual of Medical Treatment or Clinical Therapeutics. By J. Burney Yeo, M.D., F.R.C.P. In two Volumes. New and Revised Edition. (London: Cassell and Company. 1902. Price 21s. net.)

We find in this book a good deal more than is suggested by its title. It may indeed be regarded as a manual of the practice of medicine rather than of treatment alone, for although the main feature of the book lies in the full and complete manner in which treatment is dealt with, both the pathology and the symptomology of the various diseases are given with quite sufficient fulness to render the work a useful guide to the practice of medicine. The general plan of the book is that for each disease first some remarks are made in regard to etiology, then a short description is given of the pathological changes which are met with, next an account of the symptoms, which are treated with considerable fulness, while finally and more particularly comes treatment. Under the head of treatment are included not only drugs but methods, and sometimes operative proceedings The special feature of the book is that the various phases, accidents and complications of the several diseases are so dealt with as to show what is the treatment appropriate to each, and that where the use of drugs is advised formulæ for their administration are given in the text, in addition to which a list of additional formulæ is appended to nearly every chapter. From a careful perusal of the book we can have no hesitation in saying that it will prove of immense service to the practitioner, and more especially to the young practitioner, in that it supplies just what is so often lacking in modern medical teaching. So much is nowadays taken off the shoulders of the medical staff in hospital work, partly by the constant presence of trained nurses, partly by the use of pre-arranged dietaries, partly by the employment of hospital formulæ, and partly by the fact that the details of treatment are often arranged by the house physicians, and thus fail to find a place in the clinical lecture, that the therapeutical minutiæ, of which this book is so full, are apt to form a *terra incognita* to the student even to the end of his curriculum. Yet it is on a proper appreciation of the importance of these details that success in practice largely depends, and by supplying this sort of information Dr. Burney Yeo's book usefully fills a gap in medical education, in addition to providing the practitioner with an admirable guide to medical treatment in its wider aspects.

